# Improving medication adherence among persons with cardiovascular disease through m-health and community health worker-led interventions in Kerala; protocol for a type II effectiveness-implementation research-(SHRADDHA-ENDIRA)

**DOI:** 10.1186/s13063-024-08244-0

**Published:** 2024-07-02

**Authors:** Jaideep C. Menon, Denny John, Aswathy Sreedevi, Chandrasekhar Janakiram, Akshaya R, Sumithra S, Aravind M S, Mathews Numpeli, Bipin Gopal, Renjini B A, Sajeev P K, Ravivarman Lakshmanasamy, Abhishek Kunwar

**Affiliations:** 1 Adult Cardiology, AIMS, Amrita Vishwa Vidyapeetham, Kochi, India; 2 Ramaiah University of Applied Sciences, Bengaluru, India; 3 Community Medicine, AIMS, Amrita Vishwa Vidyapeetham, Kochi, India; 4 Public Health Dentistry, Amrita School of Dentistry, Amrita Vishwa Vidyapeetham, Kochi, India; 5 StJohn’s Research Institute, Bangalore, India; 6 AIMS, Kochi, India; 7 MO, DHS, Govt of Kerala, Ernakulam, India; 8 NCD, DHS, Govt of Kerala, Kerala, Thiruvananthapuram India; 9 CHC, Kalady, Kalady, India; 10 India Country Office, New Delhi, India; 11 NPO NCD, WHO India, New Delhi, India

**Keywords:** Coronary artery disease, Factorial study design, Valvular disease, Arrhythmia, Heart failure, Implementation Research, Medication adherence, Morisky Medication Adherence Scale

## Abstract

**Background:**

Cardiovascular disease (CVD) is the leading cause of mortality worldwide, and at present, India has the highest burden of acute coronary syndrome and ST-elevation myocardial infarction (MI). A key reason for poor outcomes is non-adherence to medication.

**Methods:**

The intervention is a 2 × 2 factorial design trial applying two interventions individually and in combination with 1:1 allocation ratio: (i) ASHA-led medication adherence initiative comprising of home visits and (ii) m-health intervention using reminders and self-reporting of medication use. This design will lead to four potential experimental conditions: (i) ASHA-led intervention, (ii) m-health intervention, (iii) ASHA and m-health intervention combination, (iv) standard of care. The cluster randomized trial has been chosen as it randomizes communities instead of individuals, avoiding contamination between participants. Subcenters are a natural subset of the health system, and they will be considered as the cluster/unit. The factorial cluster randomized controlled trial (cRCT) will also incorporate a nested health economic evaluation to assess the cost-effectiveness and return on investment (ROI) of the interventions on medication adherence among patients with CVDs. The sample size has been calculated to be 393 individuals per arm with 4–5 subcenters in each arm. A process evaluation to understand the effect of the intervention in terms of acceptability, adoption (uptake), appropriateness, costs, feasibility, fidelity, penetration (integration of a practice within a specific setting), and sustainability will be done.

**Discussion:**

The effect of different types of intervention alone and in combination will be assessed using a cluster randomized design involving 18 subcenter areas. The trial will explore local knowledge and perceptions and empower people by shifting the onus onto themselves for their medication adherence. The proposal is aligned to the WHO-NCD aims of improving the availability of the affordable basic technologies and essential medicines, training the health workforce and strengthening the capacity of at the primary care level, to address the control of NCDs. The proposal also helps expand the use of digital technologies to increase health service access and efficacy for NCD treatment and may help reduce cost of treatment.

**Trial registration:**

The trial has been registered with the Clinical Trial Registry of India (CTRI), reference number CTRI/2023/10/059095.

## Administrative information

Note: the numbers in curly brackets in this protocol refer to SPIRIT checklist item numbers. The order of the items has been modified to group similar items (see http://www.equator-network.org/reporting-guidelines/spirit-2013-statement-defining-standard-protocol-items-for-clinical-trials/).

**Table:** 

Title {1}	SPIRIT guidance: Descriptive title identifying the study design, population, interventions, and, if applicable, trial acronym. **Improving medication adherence among persons with cardiovascular disease through m-health and community health worker led interventions in Kerala; protocol for a Type II effectiveness-implementation research (SHRADDHA-ENDIRA)**
Trial registration {2a and 2b}.	SPIRIT guidance: Trial identifier and registry name. If not yet registered, name of intended registry. Item 2b is met if the register used for registration collects all items from the World Health Organization Trial Registration Data Set.
Protocol version {3}	SPIRIT guidance: Date and version identifier. Version 3. 23 February 2024.
Funding {4}	SPIRIT guidance: Sources and types of financial, material, and other support. Financial support from WHO, Geneva, Alliance for Health Policy and System research
Author details {5a}	SPIRIT guidance: Affiliations of protocol contributors. Jaideep C Menon, Professor, Adult Cardiology, AIMS, Kochi Denny John, Adjunct Professor, Ramaiah University of applied Sciences Aswathy S, Professor, Community Medicine Chandrasekhar J, Professor, Public Health Dentistry Akshaya R, Senior Resident, Community Medicine Sumithra S, Senior Lecturer, St John’s research Institute Aravind MS, Research Associate, Public Health, AIMS, Kochi Mathews Numpeli, CHC MO, DHS, Govt of Kerala Bipin Gopal, State nodal Officer- NCDs, Kerala Renjini BA, MO, DHS, Govt of Kerala Sajeev PK, NHM Coordinator, Kalady Ravivarman L, WHO NCD officer, India Country Office Abhishek Kunwar, NPO NCD, WHO India
Name and contact information for the trial sponsor {5b}	SPIRIT guidance: Name and contact information for the trial sponsor. Dr Sarah Rylance, Medical Officer for Chronic Respiratory Diseases, Focal point for NCD Research and Innovation World Health Organization HQ
Role of sponsor {5c}	SPIRIT guidance: Role of study sponsor and funders, if any, in study design; collection, management, analysis, and interpretation of data; writing of the report; and the decision to submit the report for publication, including whether they will have ultimate authority over any of these activities. Study sponsor does not have any role in the study design, collection, management, analysis and interpretation of data

## Introduction

### Background and rationale {6a}

Cardiovascular disease (CVD) is the leading cause of mortality worldwide, and at present, India has the highest burden of acute coronary syndrome and ST-elevation myocardial infarction (MI) [1]. A key reason for poor outcomes is non-adherence to medication. The WHO has reported that non-adherence to drugs in chronic conditions is as high as 50%, and 30% of re-admissions are related to non-compliance to medication. In its 2003 report [2], WHO states that “increasing the effectiveness of adherence interventions may have a far greater impact on the health of the population than any improvement in specific medical treatment.”

A systematic review published in 2015 on adherence to medication had eleven studies from India reporting adherence rates (using pills taken, prescribed doses taken, changes, etc.) using Morisky Medication Adherence Score (MMAS) in the range of 0–51.2% [3–8]. The factors associated with non-adherence to medications were forgetfulness, difficulty in remembering, and stopping medication upon feeling better/worse.

Various interventions have been studied to increase medication adherence for cardiovascular disease in India. These include the use of combination therapy or polypill [9–11], use of community health workers (CHW) for simplified hypertension management with the aid of a smart-phone-based electronic decision support system [12], “task shifting” interventions to CHWs for CVD risk reduction through behavioral change [13], improving adherence to drugs, lifestyle changes, and clinical risk markers in patients of acute coronary syndromes [14, 15] and use of CHWs and doctors in primary health center (PHC) to assess CVD risk with clinical decision support being provided through an m-health platform by doctors sitting remotely [16]. Studies have also identified the use of mobile technology by health workers in resource-limited settings for health delivery improvement [17]. The different studies mentioned have looked at m-health or CHWs alone to improve adherence to medication, lifestyle changes, or as a platform for treatment, with varied results.

We measured adherence in 2064 patients of coronary artery disease (CAD) the ENDIRA cohort using the MMAS-8 in the year 2019. Our results revealed poor adherence to chronic care medications in CAD patients. On an average, only 2.8 of the mandated 4 drugs (beta blocker, ACE Inhibitor /ARB, statin, and anti-platelet) were being taken by patients regularly [18]. The mean value of MMAS was 4 out of a possible 8, reflecting poor adherence [19]. A study on the feasibility of an m-health intervention in the same cohort for the prevention and management of CAD revealed that the use and ownership of mobiles was 88% (2015), 92% were willing to receive mobile health advice [19], 70% preferred voice calls over SMS, 85.9% would send self-recorded blood pressure, weight, and blood glucose to a doctor or community health worker [19]. Given that the results of our study revealed poor adherence and that use of m-health for CVD was both acceptable and feasible, the obvious next step would be in trying to improve adherence using these resources.

### Objectives {7}

#### Primary objective

1. To assess the effectiveness of using m-health and community health worker-led interventions for improving adherence to drugs in patients with cardiovascular disease using m-health and community health worker intervention individually and in combination in comparison to control group.

#### Secondary objective

(2) To assess the effects of using the interventions (m-health and community health worker-led interventions) for improving adherence to drugs among heart disease patients on implementation outcomes such as acceptability and adoption.
(3) To assess the cardiometabolic risk factors among first degree relatives of patients with heart disease

### Trial design {8}

It is a 2 × 2 factorial design trial applying two interventions individually and in combination with a 1:1 allocation ratio. Two interventions are applied individually and in combination: (i) ASHA-led medication adherence initiative comprising of home visits, and (ii) m-health intervention using reminders and self-reporting of medication use. This design will lead to four potential experimental conditions: (i) ASHA-led intervention, (ii) m-health intervention, (iii) ASHA and m-health intervention combination, (iv) standard of care.

## Methods: participants, interventions, and outcomes

### Study setting {9}

The study will be implemented in the ENDIRA (Epidemiology of Non-communicable Diseases in Rural Areas) cohort (n-114,064 individuals) which includes 2064 patients with heart disease in whom adherence to drugs for heart disease has already been assessed. The ENDIRA cohort is spread over 5 primary health centers consisting of 18 subcenters where the health details of all individuals have been recorded. In order to avoid contamination in the treatment allocation and its response, at least 10 km of distance among villages will be maintained and they will be clubbed into 4 groups.

The intervention will be implemented in Angamaly block consisting of five local self-government areas namely Mookkannoor, Kalady, Thuravoor, Karukutty, and Manjapra with a population of 18,638, 20,407, 20,475, 26,811, and 14,668 in Ernakulam district [20] in Kerala state, India, respectively.

### Eligibility criteria {10}

The study samples will consist of adult community members with diagnosis of CAD, valvular disease, heart failure, and rhythm disorders in the target areas who provide informed consent.

#### Eligibility criteria

- Diagnosed case of CAD who have received treatment for MI/STEMI/UA or diagnosed using a coronary angiogram or CT coronary angiogram or have undergone revascularization and are on medications.
- Other cardiovascular cases such as rhythm disorders, valve disorders, and heart failure identified as pumping disorders by the community will also be a part of the study. Male or female aged 18 years or more will be considered.
- Resident of village during the baseline survey.
- Has no plans to migrate in next 12 months from the date of initiation of intervention.

#### Exclusion criteria

- Persons who are bedridden and are unable to answer the questions.
- Pregnant or lactating mothers
- Individuals with cognitive impairment

### Who will take informed consent? {26a}

Informed consent will be taken by the accredited social health activist of the area who will be collecting the data. The data collection will be through an application called SHRADDHA (which means care). The participant’s digital signature will be obtained on the tablet.

### Additional consent provisions for collection and use of participant data and biological specimens {26b}

 Blood samples will be collected to assess random blood sugar and HbA1c among cardiac patients with type 2 DM after obtaining consent. These samples will be tested using point-of-care devices and will not be stored. We will request consent for review of participants’ medical records, and for the collection of blood samples to assess random blood sugar and HbA1c among the cardiac patients with type 2 diabetes. But this trial does not involve collecting biological specimens for storage.

## Interventions

### Explanation for the choice of comparators {6b}

Results of our study revealed poor adherence and that use of m-health for CVD was both acceptable and feasible. Various interventions have been studied to increase medication adherence for cardiovascular disease in India such as use of combination therapy or polypill, use of community health workers (CHW) for simplified hypertension management with the aid of a smart phone-based electronic decision support system, so we decided to use factorial study design where study units would be assigned to ASHA and no ASHA group. Following this they would be assigned to m-health and no m-health group. Thus, there are four arms to the study: namely ASHA, ASHA and m-health, m-health, and standard of care.

### Intervention description {11a}

The intervention content is prepared after discussion with the stakeholders such as ASHAs, Medical Officers, and patients. Qualitative data would be obtained from unstructured or semi-structured interviews exploring the individual’s understanding of the use of medicines, potential obstacles and incentives to adherence, useful strategies to improve adherence. Interview guide for In-Depth-Interviews and Key-Informant Interviews will be developed after a thorough literature search. In-Depth Interviews will be done with the participants and their relatives to identify individual’s understanding of the use of medicines, potential obstacles and incentives to adherence, useful strategies to improve adherence, and other questions spontaneously raised during the interview. For Key-Informant Interviews, Health care providers such as doctors, the multipurpose health worker, ASHAs, and pharmacists (about 10) will be interviewed till saturation Is reached. Focus group discussions (FGD) will be conducted among adherent CVD patients and nonadherent CVD patients. About 3-4 such FGD will be conducted till data saturation is reached. This will be repeated at endline.

Community health worker directed visits to the house of the patient, where they will explain the use of drugs and the various roles of the different classes of drugs along with taking a pill count and giving health advice and counselling with a PowerPoint on a tablet. The frequency of visits is twice a month for the first 3 months, and once a month for the next 3 (11 visits in all). A schedule of visits with the areas to be highlighted in each visit such as diet, physical activity, tobacco, and alcohol will be prepared and given.

Before the commencement of the intervention training, sessions for community health workers (ASHAs) in the intervention arm will be conducted. This will comprise of three sessions of 6 h each and would include curriculum-based training modules on CVD, HTN, diabetes, dyslipidemia; awareness of the role each of the 4 classes of drugs in AS-CVD plays in secondary prevention; sensitization to the role of adherence in preventing recurrence; sensitization to the side effects of the drugs and counselling skill training. Role of lifestyle changes such as diet, physical activity, tobacco, and alcohol will also be carried out.

#### m-health

The envisaged m-health platform is a two-way system through which messages or jingles (audio clips) could be passed back and forth between the care provider (ASHA, Research assistant, or doctor) and the recipient (patient). Individual patient details gathered and entered on a Tab PC get stored on a central server. The data is anonymized and coded individually, with a 12-digit UID. In clusters where the m-health intervention is planned, individual patients could download and activate an already developed App, which is a free download from the Google play store [Ente app (my app)]. The individual patient would be able to access his personal health record as entered, by way of the App. This App would serve as a two-way channel of communication between the patient and caregiver. In the other clusters, individual patients could download their personal data and the App, with the communication channel remaining blocked.

Bi-monthly reminders via text or audio messages and weekly reminders on taking medicines at the time of a scheduled dose by way of a beep or tune and health advice by way of messages are sent for the first 3 months, followed by monthly reminders of text messages the next 3 and weekly drug reminder tunes.

#### Community health worker and m-health

Health worker (ASHA)-directed visits to the house of the patient, where they will explain the use of drugs and the various roles of the different classes of drugs along with taking a pill count and giving health advice and counselling. The frequency of visits is twice a month for the first 3 months, and once a month for the next 3. In addition, bi-monthly reminders via text or audio messages and weekly reminders on taking medicines at the time of a scheduled dose by way of a beep or tune and health advice by way of messages are sent for the first 3 months, followed by monthly reminders of text messages the next 3 and weekly drug reminder tunes.

*Standard of care (SoC)* is patient-initiated physician visit with health advice and treatment as prescribed by the treating doctor.

In all the groups, the patients can visit the doctor in case of any need or emergency.

 After completion of baseline survey in all clusters, intervention will be implemented in intervention clusters for 6 months. All the participants in the intervention and control arms will be permitted to use standard treatment for CVD. Community health worker-directed visits to the house of the patient, where they will explain the use of drugs and the various roles of the different classes of drugs along with taking a pill count and giving health advice and counselling. The frequency of visits is twice a month for the first 3 months, and once a month for the next 3 (9 visits in all). Table 1 shows the timepoint for the intervention implementation.

### Criteria for discontinuing or modifying allocated interventions {11b}

This is not applicable as the intervention is to improve medication adherence, so there will be no special criteria for discontinuing or modifying allocated intervention.

### Strategies to improve adherence to interventions {11c}

The various interventions are for the improvement of adherence as measured by the Morisky Medication Adherence scale [21].

### Relevant concomitant care permitted or prohibited during the trial {11d}

Relevant concomitant care is permitted.

### Provisions for post-trial care {30}

This is a non-pharmacological intervention; therefore, there are no specific post trial care provisions. 

### Outcomes {12}

#### Primary, secondary, and other outcomes

The primary outcome is the adherence of patients as measured by Morisky adherence scale [21] at the beginning of the study, midterm, and at the end. The secondary outcomes include Quality of Life (EuroQOL) [22], blood pressure, random blood sugar, HbA1c among the cardiac patients with type 2 diabetes, mortality events, and other unintended outcomes will also be recorded. The analysis will include change from baseline. Adherence is chosen as the main outcome as the objective is to study the impact of the various interventions singly and in combination on adherence in comparison to standard of care. Various symptoms, such as dyspnea, fatigue, edema, difficulty sleeping, depression, and chest pain associated with CVD limits activities of daily life [23]. Therefore, it is important to measure the quality of life before and after the intervention. Metabolic control can result from better adherence to medication and a better awareness of the importance to adhering to medication. Therefore, meeting targets of blood pressure, blood sugar levels, and HbA1c will be considered as secondary outcomes.

### Participant timeline {13}

**Table 1:** Timeline of activities

**Study period**	**Enrollment**	**Allocation**	**Post allocation**						Close-out
			**Month 1**	**Month 2**	**Month 3**	**Month 4**	**Month 5**	**Month 6**	
**Enrollment**									
Eligibility screen	♦								
Informed consent	♦								
Allocation		♦							
**Interventions**									
Intervention A			♦	♦	♦	♦	♦	♦	
Intervention B			♦	♦	♦	♦	♦	♦	
Intervention C			♦	♦	♦	♦	♦	♦	
Intervention D			x	x	x	x	x	x	
**Assessments**									
Baseline assessment	♦	♦							
Primary outcome									♦
Secondary outcome									♦

### Sample size {14}

Based on the learnings from the previous study, the rate of missing data due to electronic data collection will be low.

#### Phases 1 and 2: planning and baseline evaluation

The process of developing the intervention will start with the development of the initial concepts based on the available literature and interaction with healthcare professionals working in the rural areas.

## Baseline study

*Selection and training of team*: The team will deliver the training to the selected project coordinator and the field staff. Field staff (part-time) will be recruited by the investigators on the advice of village head and/or NCD clinic in-charge. He/she should be a member of community preferably the accredited social health activist with an interest in health care and community, willingness to learn, and leadership qualities. A strong commitment to work in the community will be identified as an important criteria for the selection of all the team members. After a sensitization session of the data collectors/field staff, they will be asked to prepare a list of persons with cardiovascular disease including coronary artery disease, valvular disease, arrhythmias, and heart failure. Hands on sessions to download the App and collect data will be provided.

In Phase 2, baseline evaluation will be initiated in the study areas after obtaining the ethics committee approval. Written informed consent will be obtained from the study participants. Participants will receive a participant information sheet (PIS) outlining the rationale for the study, details on interventions, the steps, and protocols to be followed throughout the study, potential side effects and risks, benefits, a confidentiality statement, the option to withdraw from the study at any time, and the investigators’ contact information. The baseline survey performed by ASHAs will be done through a survey app called SHRADDHA. The variables collected would include (1) basic demographic information, including age, income, gender, marital status, religion, and occupation; (2) lifestyle-related factors such as physical activity, tobacco use, and alcohol consumption, dietary factors intake of fruits and vegetables, cooking oil and red meats; (3) disease details including for diabetes, hypertension, dyslipidemia, stroke and CAD, COPD, and surgeries; and (4) current medications. Questions will be explained to each participant to help them get familiar with the contents, instructions for filling them out will be given, and the responses will be recorded. On the home visit, the Field staff/ASHA will also record height and weight, measure sugars with a glucometer, and take a photo of the most recent prescription. All of this will be recorded in the app. Glycosylated hemoglobin will also be measured among the cardiac patients with diabetes using the point of care device called Lumira Dx.

*Sample size*: Sample size was estimated assuming an improvement of 10% in medication adherence at the end of a 6-month period in either m-health or community health worker-led intervention compared to control group. This 10% improvement will lead to an effect size of 0.4 units in medication adherence through m-health or community health worker-led intervention and an effect size of 0.8 units in combined intervention (m-health and community health worker-led intervention) compared to the control group. The 10% was an assumption considering that large differences are not possible in a community setting and was based on another community-based study which has also used 10% improvement of adherence score [24].

To observe a difference of 0.4 units in the medication adherence between study groups, with a standard deviation of 1.8, 5% level of significance (adjusted for multiple comparison) and 80% power, the sample size needed will be 238 participants in each of the study groups. After accounting for a design effect (cluster effect) and 10% attrition, the number of participants required per group will be 393, a total of 1572 participants.

### Recruitment {15}

Working through the public health system, keeping in mind the proximity of the ASHAs to the community, it is expected that adequate participant enrolment can be achieved. Monitoring and supervision by the project team will assist in timely completion. The time period of recruitment is from February to May, 2024. After the recruitment, the randomization will be done and intervention will be administered for 6 months. Expected to finish by November 2024 and endline assessment in December 2024.

## Assignment of interventions: allocation

### Sequence generation {16a}

Allocation of intervention and sequence generation will be as follows. Codes will be randomly assigned to the four interventions (ASHA, m-health, ASHA + m-health, and control groups) namely A, B, C, and D. In the next step, randomization list will be generated using RANDOM ALLOC software. Eighteen subcenters will be randomized into 4 study groups (A, B, C, and D) using different permutations of ABCD. Each subcenter will be allocated random numbers ranging from 1 to 18 using random number generators and random shuffling of this number. Interventions will be allocation to the subcenters in the sequence of random shuffled numbers as per the randomization list.

### Concealment mechanism {16b}

It will be an open-label trial as concealment is not possible. However, study site allocation will be done only after completing baseline assessment and agreements with sites to participate.

### Implementation {16c}

The allocation sequence will be generated by the Statistician, enrolment will be by the Field coordinator and the Field coordinator will assign subcenter as it is a cluster randomized trial.

## Assignment of interventions: blinding

### Who will be blinded {17a}

Data analysts will be blinded. The ASHA workers, patients, and outcome assessors are not blinded.

### Procedure for unblinding if needed {17b}

In this study, the ASHA workers, patients, and outcome assessors will not be blinded. Only the data analysts will be blinded. The data analyst will be unblinded if there are any outlier biochemical values which requires immediate action so that the patient can be intimated.

## Data collection and management

### Plans for assessment and collection of outcomes {18a}

The primary outcome adherence will be measured by Morisky 8-item adherence questionnaire which has been validated in various countries including India and in various disease conditions. The eight-item Morisky Medication Adherence Scale (MMAS-8) is a structured self-report measure of medication-taking behavior that has been widely used in various cultures [25–27].It has a maximum score of 8.

The quality of life will be measured by the EuroQol five‐dimensions – 3‐level (EQ5D) which is a versatile quality of life (QOL) instrument with five dimensions (mobility, self‐care, usual activities, pain/discomfort, and anxiety/depression) and a visual analog scale. The questionnaire has also been found to be valid and reliable in various disease conditions including cardiovascular and cancer in India and neighboring countries [28, 29].Random blood sugar among the patient and family member will be measured by the ASHA as per standard methods using a glucometer. Blood pressure will also be measured using the electronic Blood pressure will be recorded with the OMRON HEM 7124 automatic blood pressure monitor (Shimogyo-ku, Kyoto, Japan) by measuring upper arm BP. A laboratory technician will measure Glycosylated Haemoglobin using the Lumira Dx point of care device.

Real-time data entry will be monitored, and wherever there are difficulties with using the app, support will be provided by the field coordinator.

### Plans to promote participant retention and complete follow-up {18b}

All efforts will be made to retain all participants in the study. As they are also part of the earlier ENDIRA study, there is a good rapport with the study group, local self-government, and frontline health workers. Loss to follow-up may result from migration to their children’s places of living or death or for other reasons. The characteristics of the patients who drop out will be recorded and compared to those who are in the study.

### Data management {19}

As the data is collected through the SHRADDHA app, the data will be exported to excel and checked for completion each day. According to the data collected, feedback, and monitoring will be done to ensure correct and complete entries. Duplicate entries will be checked for and removed.

### Confidentiality {27}

The data of the patients will be anonymized, and each patient will be assigned a unique id. From the app de-identified anonymized data will be stored in Excel. This will be stored confidentially before, during, and after the trial.

### Plans for collection, laboratory evaluation, and storage of biological specimens for genetic or molecular analysis in this trial/future use {33}

In this study, blood samples will be collected to assess random blood sugar and HbA1c. These tests are done using point-of-care devices. The blood samples will not be stored in the current trial.

## Statistical methods

### Statistical methods for primary and secondary outcomes {20a}

Several models will be run to test for the main outcomes, implementation outcomes, and related research questions. Mixed linear and logistic effects models as appropriate will be used to identify differences between the groups (ASHA, ASHA and m-health, m-health, control group), where random effects will be used for the clusters and fixed effects will be used for effects of ASHA workers and of m-health. The primary dependent variable in the models will be change in adherence measured by the Morisky scale. Models will also be fitted for the secondary outcomes such as change in blood pressure, random blood sugar, Hba1c levels, and quality of life. Subsequently, covariates such as age, sex, and co-morbidities will be added to the models to adjust for potential confounders.

### Interim analyses {21b}

In this study, the intervention is done to improve the medication adherence through health education by ASHA workers, m-health, or both. Since the risk due to the intervention is minimal or none, interim analysis, and stopping guidelines have not been prescribed by the ethical committee and therefore there will not be any stopping guidelines.

### Methods for additional analyses (e.g., subgroup analyses) {20b}

Subgroup analysis will not be carried out. However, for the primary and secondary outcome variables, covariates such as age, sex, and co-morbidities will be considered as potential confounders in the mixed effect model analysis.

### Methods in analysis to handle protocol non-adherence and any statistical methods to handle missing data {20c}

Nonadherence will be managed by the intention to treat analysis and if there are too many missing data, imputations will be considered. Mixed method analysis will be considered for intention to treat analysis. Also depending on the percentage of data missingness and assumption for data missing in the study variables, appropriate missing data imputation technique will be used.

### Plans to give access to the full protocol, participant-level data, and statistical code {31c}

Full protocol can be given. Full dataset can be given with the permission of the Institution, WHO, and Government.

## Oversight and monitoring

### Composition of the coordinating center and trial steering committee {5d}

There is only one site for the study; therefore, the coordinating and steering committee will be situated at the site. The coordinating center is the Community Health Centre (CHC). The ASHA’s work is coordinated through the CHC by the National Health mission coordinators. The trial steering committee (TSC) monitors recruitment, communicates, and provides conflict resolution and timely advice. They meet every 6 weeks. Local organization and implementation is taken care of by the NHM coordinators and a responsible person reporting to the Principal Investigator from the Project management group. Trainings and other group meetings are conducted by the project management group. Consent is obtained by the ASHA. Periodic meetings are conducted by the Project management group (investigators) team to monitor progress. The stakeholder groups are apprised of the progress of the trial, role of intervention, and its possible benefit.

### Composition of the data monitoring committee, its role and reporting structure {21a}

This study is measuring adherence which is a low-risk intervention; therefore, a data monitoring committee is not required. The project management group meets every 2 weeks. The Trial Steering Group and the independent Data Monitoring and Ethics Committee meet to review conduct throughout the trial period. 

### Adverse event reporting and harms {22}

As this study is measuring adherence of an intervention, no adverse events or serious adverse events and harms from the intervention are anticipated. But if there are any, they will be reported to relevant regulatory bodies such as Project management group, Trial steering Committee, District Health Authority, and Ethics Committee. Trial deviations will be reported to the ethical committee.

### Frequency and plans for auditing trial conduct {23}

The meetings of the Project management group, Trial Steering group, independent data monitoring, and Ethics Committee periodically will also serve to audit the trial.

### Plans for communicating important protocol amendments to relevant parties (e.g. trial participants, ethical committees) {25}

Before the start and at the start, there have been some minor modifications which has been updated to the ethical committee and subsequently uploaded in the CTRI.

### Dissemination plans {31a}

The results of the study will be published in standard journals. Social media and Stakeholder workshops will be used to disseminate the findings. A lay summary  will be shared with all participants.

## Discussion

The present study will promote much needed research and innovation for increasing adherence among patients with cardiovascular disease. The effect of different types of intervention alone and in combination will be assessed using a cluster randomized design involving 18 subcenter areas. This factorial cluster randomized controlled trial will benefit by increasing the drug adherence for NCD using m-health platform and frontline health workers. The trial will explore local knowledge, perceptions and empower people by shifting the onus onto themselves for their medication adherence.

The proposal is aligned to the WHO-NCD aims of improving the availability of affordable basic technologies and essential medicines, improving adherence for non-communicable diseases (NCDs). It also aligns to WHO-NCD aim of training the health workforce and strengthening the capacity of health systems, particularly at the primary care level, to address the control of NCDs. The proposal also helps expand the use of digital technologies to increase health service access and efficacy for NCD treatment and may help reduce the cost of treatment.

The proposal helps implementation of WHO-PEN protocol for Self-Care guidelines including utilizing frontline health workers in improving self-care in patients of heart disease, counselling to improving adherence and self-care, considering patients’ beliefs and concerns about drugs and their effect. The research is also aligned to the WHO-HEARTS package, both by way of A&T of the HEARTS where A- consists of information on CVD medicine and technology procurement, quantification, distribution, management, and handling of supplies at facility level. T- consists of guidance and examples on team-based care and task shifting related to the care of CVD. The research is also aligned with the Sustainable Development Goals (SDG) goal in relation to NCD of reducing by one third premature mortality from non-communicable diseases through prevention and treatment.

There are significant expected implementation challenges to note. First, the trial involves working with the primary clinics providing NCD screening and detection services, and building an effective partnership with the state government of Kerala where the project will be implemented will be crucial for its success. Second, medication nonadherence for patients with chronic diseases is extremely common with 40–50% of patients prescribed medications for management of diabetes and hypertension [30]. There exist treatment-related barriers, such as treatment complexity, side effects (or fear of side effects), inconvenience, cost, and time, and other barriers such as poor practitioner-patient relationship, aspects of which are beyond the scope of the intervention [30].

If successful, the medication adherence intervention, using m-health and ASHAs, has the potential to constitute evidence-based practice for improving medication adherence for CVD in India, and in similar developing countries.

## Trial status

The current protocol is version 3 dated 23–02-2024. The recruitment began on November 30, 2023 and is expected to be complete by May 30, 2024. The submission has been delayed due to unavoidable circumstances such as elections and heatwave.

## Data Availability

The investigators will have access to the final data set. There are no contractual agreements which limit access to investigators. The investigators in the field collect the data and the data is with them. Any data required to support the protocol can be supplied on request.

## Abbreviations

ACE: Angiotensin-converting enzyme
ARB: Angiotensin II receptor blocker
ASHA: Accredited social health activist
CAD: Coronary artery disease
CHW: Community health workers
COPD: Chronic obstructive pulmonary disease
cRCT: Cluster randomized control trial
CT: Computed tomography
CTRI: Clinical Trials Registry—India
CVD: Cardiovascular diseases
ENDIRA: Epidemiology of Non-Communicable Diseases in Rural Areas
HT: Hypertension
LMIC: Low-middle-income countries
m-health: Mobile Health
MI: Myocardial infarction
MMAS: Morisky Medication Adherence Score
NCD: Non-communicable diseases
PHC: Primary health center
QoL: Quality of Life
ROI: Return on investment
SDG: Sustainable Development Goals
SMS: Short Message Service
STEMI: ST elevation myocardial infarction
UA: Unstable angina
WHO: World Health Organization
