# Correction: Improving medication adherence among persons with cardiovascular disease through m-health and community health worker-led interventions in Kerala; protocol for a type II effectiveness-implementation research-(SHRADDHA-ENDIRA)

**DOI:** 10.1186/s13063-024-08323-2

**Published:** 2024-07-09

**Authors:** Jaideep C. Menon, Denny John, Aswathy Sreedevi, Chandrasekhar Janakiram, R Akshaya, S Sumithra, M. S Aravind, Mathews Numpeli, Bipin Gopal, B. A Renjini, P. K Sajeev, Ravivarman Lakshmanasamy, Abhishek Kunwar

**Affiliations:** 1 Adult Cardiology, AIMS, Amrita Vishwa Vidyapeetham, Kochi, India; 2 Ramaiah University of Applied Sciences, Bengaluru, India; 3 Community Medicine, AIMS, Amrita Vishwa Vidyapeetham, Kochi, India; 4 Public Health Dentistry, Amrita School of Dentistry, Amrita Vishwa Vidyapeetham, Kochi, India; 5 StJohn’s Research Institute, Bangalore, India; 6 AIMS, Kochi, India; 7 MO, DHS, Govt of Kerala, Ernakulam, India; 8 NCD, DHS, Govt of Kerala, Kerala, Thiruvananthapuram India; 9 CHC, Kalady, Kalady India; 10 India Country Ofce, New Delhi, India; 11 NPO NCD, WHO India, New Delhi, India

**Correction: Trials 25, 437 (2024)**

**https://doi.org/10.1186/s13063-024-08244-0**

Following the publication of the original article [1], we were notified that the 12th author’s name was incorrectly spelled.

Originally published author name: Ravivarman Lakshmanaswamy

Correct name: Ravivarman Lakshmanasamy

The original article was corrected.
