# Correction: Improving medication adherence among persons with cardiovascular disease through m-health and community health worker-led interventions in Kerala; protocol for a type II effectiveness-implementation research-(SHRADDHA-ENDIRA)

**DOI:** 10.1186/s13063-025-08789-8

**Published:** 2025-03-11

**Authors:** Jaideep C. Menon, Denny John, Aswathy Sreedevi, Chandrasekhar Janakiram, Akshaya R, Sumithra S, Aravind M. S, Mathews Numpeli, Bipin Gopal, Renjini B. A, Sajeev P. K, Ravivarman Lakshmanasamy, Abhishek Kunwar

**Affiliations:** 1 Adult Cardiology, AIMS, Amrita Vishwa Vidyapeetham, Kochi, India; 2 Ramaiah University of Applied Sciences, Bengaluru, India; 3 Community Medicine, AIMS, Amrita Vishwa Vidyapeetham, Kochi, India; 4 Public Health Dentistry, Amrita School of Dentistry, Amrita Vishwa Vidyapeetham, Kochi, India; 5 StJohn’s Research Institute, Bangalore, India; 6 AIMS, Kochi, India; 7 MO, DHS, Govt of Kerala, Ernakulam, India; 8 NCD, DHS, Govt of Kerala, Kerala, Thiruvananthapuram India; 9 CHC, Kalady, Kalady India; 10 India Country Ofce, New Delhi, India; 11 NPO NCD, WHO India, New Delhi, India

**Correction: Trials 25, 437 (2024)**

**https://doi.org/10.1186/s13063-024-08244-0**

Following the publication of the original article [1], we were notified that the Funding section needs to be updated.

**Originally published Funding declaration:**

*“Funded by WHO NCD Division and NCD Alliance, Geneva.*

*WHO Reference 2023/1376413–0.*

*The funding body does not have a role in the design, data collection, analysis, and interpretation of data.”*

**Correct Funding declaration:**

*"This research study is supported by the Alliance for Health Policy and Systems Research and World Health Organization (WHO) Department of Noncommunicable Diseases, Rehabilitation and Disability.*

*The Alliance is able to conduct its work thanks to the commitment and support from a variety of funders. These include our long-term support from the Swedish International Development Cooperation Agency (Sida) and the Norwegian Agency for Development Cooperation (Norad), as well as designated funding for specific projects within our current priorities. For the full list of Alliance donors, please visit* https://ahpsr.who.int/about-us/funders *".*

The original article was corrected.
